# Effects of International Labour Migration on the Mental Health and Well-Being of Left-Behind Children: A Systematic Literature Review

**DOI:** 10.3390/ijerph17124335

**Published:** 2020-06-17

**Authors:** Khatia Antia, Johannes Boucsein, Andreas Deckert, Peter Dambach, Justina Račaitė, Genė Šurkienė, Thomas Jaenisch, Olaf Horstick, Volker Winkler

**Affiliations:** 1 Heidelberg Institute of Global Health, Heidelberg University Hospital, Im Neuenheimer Feld 130.3, 69120 Heidelberg, Germany; johannes.boucsein@uni-heidelberg.de (J.B.); a.deckert@uni-heidelberg.de (A.D.); peter.dambach@web.de (P.D.); thomas.jaenisch@urz.uni-heidelberg.de (T.J.); olaf.horstick@uni-heidelberg.de (O.H.); volker.winkler@uni-heidelberg.de (V.W.); 2 Department of Public Health, Institute of Health Sciences, Faculty of Medicine, Vilnius University, M. K. Čiurlionio str. 21, LT-03101 Vilnius, Lithuania; justina.racaite@mf.vu.lt (J.R.); gene.surkiene@mf.vu.lt (G.Š.)

**Keywords:** left-behind, children, labour migration, parent, mental health, well-being, transnational families

## Abstract

Labour migration is a challenge for the globalised world due to its long-term effects such as the formation of transnational families. These families, where family members of migrant workers are “left-behind”, are becoming a common phenomenon in many low- and middle-income countries. Our systematic literature review investigated the effects of international parental labour migration on the mental health and well-being of left-behind children. Following the PRISMA guidelines, we performed searches in PubMed, PsychINFO, Web of Science, Cochrane Library and Google Scholar, resulting in 30 finally included studies. We found that mental health and well-being outcomes of left-behind children differed across and sometimes even within regions. However, only studies conducted in the Americas and South Asia observed purely negative effects. Overall, left-behind children show abnormal Strengths and Difficulties Questionnaire scores and report higher levels of depression and loneliness than children who do not live in transnational families. Evidence from the studies suggests that gender of the migrant parent, culture and other transnational family characteristics contribute to the well-being and mental health of left-behind children. Further research utilising longitudinal data is needed to better understand the complex and lasting effects on left-behind children.

## 1. Introduction

Worldwide, 272 million people were classified as international migrants with the largest proportions coming from Asia (41%) and Europe (23.7%) [1,2]. Parental labour migration is a common phenomenon in South and South East Asian, African and Eastern European countries, causing many children to be left behind [3,4,5]. For example, 27% of all children in the Philippines are considered to be left-behind [6], 37% in Ghana [7], 36% in Moldova and 39% in Georgia [8]. Often, international migration is temporary and does not involve all family members. In 2017, 58.3% of all international migrants went abroad to work, leaving behind children, parents and spouses [9]. In the same year, migrants originating from developing countries sent USD 466 billion of remittances, 9.4% of the global gross domestic product [1,9]. Although labour migration reduces unemployment and increases economic efficiency in migrants’ home countries, transnational families are formed with the particularly vulnerable groups of left-behind children (LBC) and the elderly.

UNICEF [10] defines all children who suffer from inequalities in health, education and well-being as “left-behind”. However, when it comes to LBC of migrants, there is no unified definition [11]. Scholars from China defined the term LBC in the context of parental labour migration [11]. Available data from rural–urban migration suggest that parental migration has more negative than positive effects on LBC [12]. Previous research on labour migration and LBC has demonstrated that family characteristics and arrangements, caregiving practice, culture and the gender of the migrant parent play an important role [13,14,15]. However, most of the evidence comes from China, where 22% (61 million) of all children are affected by migratory separation [12,16]. Chinese labour migration happens mainly within the country as migration between rural and urban areas and is therefore not applicable to the global context [17]. With regard to the duration of parental migration, an absence of six months or longer seems problematic [11].

Generally, little attention is given to international labour migration and its effects on LBC, even though international migration is usually characterised by longer separation periods compared with internal migration. To our knowledge, this is the first systematic literature review investigating the effects of international labour migration on LBC’s mental health and well-being.

## 2. Materials and Methods

This study follows the principles of Preferred Reporting Items for Systematic Reviews and Meta-Analyses (PRISMA) [18]. Database searches, title, abstract and full-text screening as well as data extraction were independently performed by the authors KA and JB. Any disagreement was solved between all authors.

### 2.1. Search Strategy and Eligibility Criteria

To identify relevant articles, we performed a comprehensive search of the literature in English on PubMed, Web of Science, PsychINFO, Cochrane Library and Google Scholar for studies investigating the effects of international parental labour migration on LBC. The searches were performed up to April 2020 using the following broad search terms for all databases: (international migration OR transnational families OR left-behind) AND children AND (health OR well-being OR education). We modified the search strategy for Google Scholar by screening stepwise 50 results until after 200 results where no further relevant hits were found. Additionally, we screened the reference lists of included studies and searched for gray literature using the following websites: OpenGrey [19] and GreyLit [20].

We included studies on children with at least one of their parents working abroad for six months or longer. Main outcome measures were mental health and well-being related to mental health (defined as anxiety, depression, behavioural changes, self-reported happiness, life satisfaction and loneliness).

We excluded studies due to the following reasons: participants were older than 21, investigated internal migration and descriptive studies without a control group. Further, we did not consider case reports, qualitative studies and opinion papers.

### 2.2. Data Extraction

We adapted the form of the Cochrane Collaboration Public Health Group [21] and extracted from each study the following information: authors, journal and publication date, country and type of study, aims and objectives, sampling techniques and dates of data collection, sample size and age of participants, exposures and outcomes including outcome measures, key conclusions, limitations and recommendations. Additionally, we extracted available information on variances in socioeconomic status and its effect on the mental health and well-being of LBC.

### 2.3. Risk of Bias, Quality Assessment

We used the U.S. Department of Health and Human Services Quality Assessment Tools [22] to assess the risk of bias of all included studies. Considering the different study designs, we applied the following three tools accordingly: (i) Tool for Observational Cohort and Cross-Sectional Studies, (ii) Tool for Case-Control Studies, and (iii) Quality Assessment of Systematic Reviews and Meta-Analyses. We assessed the studies for criteria related to research questions and objectives, the sample size, the selection of participants and controls, clarity, validity and reliability of outcome measures, confounding variables as well as statistical analysis. Tool (i), (ii) and (iii) consist of 14, 12, and 8 items, respectively. We considered studies scoring below 50% of the respective maximum score as having a high (C) risk of bias. Those scoring between 50% and 70% having a moderate (B) and those above 70% having a low (A) risk of bias.

### 2.4. Analysis

We analysed all data according to the following information: country of origin, dataset used, methodology, outcome measures and gender aspects. Additionally, we compared available quantitative outcome measures, i.e., Strengths and Difficulties Questionnaire (SDQ) and Total Difficulties Score (TDS). The SDQ is a screening tool to evaluate the mental health of children aged 4–16 [23]. It consists of 25 items grouped into 5 different scales such as: emotional symptoms, conduct problems, hyperactivity, peer relation problems and pro-social behaviour. Each scale consists of 0–10 scores while 0 indicates the absence of problems. The SDQ is the most commonly used, validated tool for child mental health assessment and has been translated into more than 60 languages [24]. The TDS is derived from the SDQ [25]. It sums up all SDQ scales, except the pro-social scale; hence, it ranges from 0–40 and higher scores indicate higher levels of psychological distress [24]. If possible, we calculated means, fractions, odds ratios and their simple confidence intervals from the given results using the statistical computing software R (version 3.5, R Core Team; R Foundation for Statistical Computing, Vienna, Austria). Considering the wide range of study characteristics in terms of study design and study population, we decided to forgo a formal meta-analysis.

## 3. Results

### 3.1. Study Selection

**Figure 1:**
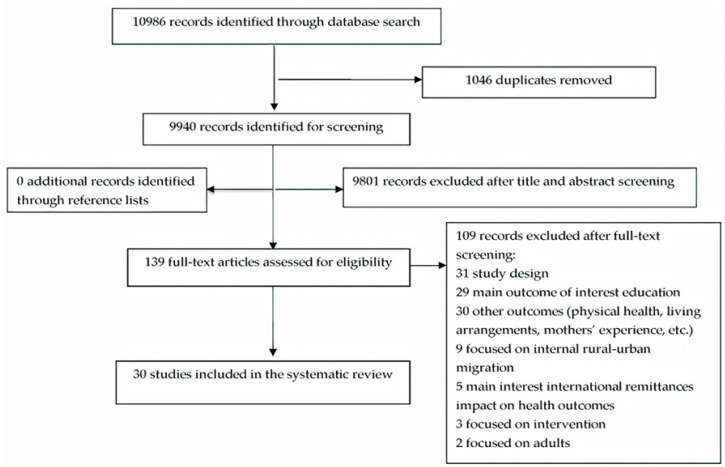
Study selection process.

From all databases searched, we identified 10,986 records of which 9940 were non-duplicates. After title and abstract screening, we retrieved full-texts from 139 records. During the full-text screening, we excluded qualitative, ethnographic studies, discussion, opinion papers, editorials, conference abstracts, non-systematic literature review papers and technical reports. Further, we excluded studies focusing on internal migration, adults and outcomes other than mental health and well-being. The majority of excluded full-texts aimed at education, physical health, living arrangements, parents’ experiences, materialism, gratitude and the impact of remittances. Finally, 30 articles were included in the analysis. Figure 1 describes the selection process and reasons for exclusion. No additional relevant studies were obtained from the reference screening and grey literature search.

### 3.2. Study Characteristics

Table A1 shows the main characteristics of all included studies, along with the risk of bias assessment and an ID number which is used to refer to individual studies.

#### 3.2.1. Study Design

We identified 20 descriptive studies that included children of non-migrant parents as control groups [3,4,8,13,24,26,27,28,29,30,31,32,33,34,35,36,37,38,39,40]. Two studies used longitudinal data [7,41]. Furthermore, we found seven mixed methods studies reporting quantitative results [5,42,43,44,45,46,47].

#### 3.2.2. Geographical Context

Eleven studies were conducted in South East Asia, some covering more than one country (five Indonesia, seven the Philippines, three Thailand, three Vietnam), three in South Asia (one India, two Sri Lanka), five in the Americas (one Jamaica, three Mexico, one Peru), seven in Eastern Europe (three Georgia, one Lithuania, three Moldova, two Romania) and five in Africa (one Angola, four Ghana, two Nigeria). Two studies had a cross-regional focus covering Ethiopia, Ghana, India, Peru and Vietnam.

#### 3.2.3. Participant Characteristics

Characteristics such as study setting, outcome measures and tools used varied considerably across studies. The main study groups were children of migrant mothers, children of migrant fathers and children of both migrant parents. Most of the studies collected data either at home or at school from the children themselves or from their caregivers using interviews or SDQ developed for parents. Several studies targeted only specific age groups such as adolescents [5,46]. For data collection, most of the authors used stratified sampling to identify schools or households with LBC.

#### 3.2.4. Outcome Measures

The most commonly used tools for the mental health assessment were the SDQ [26,27,33,40] and the TDS [24,30,38]. Other standardised tools included the Child Behavior Check List (CBCL-S), the Socio-demographic, Risk-factors Information Questionnaire (SDRIQ) [45], the Homesickness Questionnaire (HQ) [31], Short Mood and Feelings Questionnaire (SMFQ) [32] and a cognitive ability test [41]. Along with standardised tools, some authors used self-reported mental health and well-being measures, such as self-evaluated health, happiness, life satisfaction and school enjoyment [3,7,13,24,28,37,38,46]. Additionally, some studies investigated vulnerability, loneliness, being subject to bullying, involvement in conflicts and other behavioural outcomes. In some studies, authors used scales such as the Social Anxiety and Loneliness Scale [21], Anger Expression Scale for Children (AESC) [32], Parent–Child Relationship Schema Scale (PCRSS), Social Anxiety Disorder Dimensional Scale (SADDS) [39] and Multi-dimensional Scale of Perceived Social Support (MSPSS) [40]. Other tools included multi-dimensional well-being indexes consisting of the following six domains: education, physical and emotional health, housing, protection and communication access [4]. Some authors calculated a household wealth index to determine the socio-economic status of transnational households [27]. In other studies, authors examined well-being outcomes of LBC by transnational family arrangements [7,24,27,30,44]. In some studies, remittances, caregiver’s involvement and child-care arrangement were used as a measure of transnational family characteristics that may contribute to the health and well-being of LBC [3,7,24,29,44].

Most authors performed a descriptive analysis to compare the results of children with different migration profiles and calculated percentages, means with standard deviations, Pearson’s chi-squared-test or t-test. Some investigators used bivariate and multivariate models [24,27,29,30,32,33,35,41], e.g., multivariate logistic regression [27,29,33], multiple regression [24,30,32], child fixed-effects estimator, regression specifications [41], sequential quantile regression [35] and multiple analyses of variance (MANOVA) [32].

#### 3.2.5. Projects

Eleven manuscripts used nationally representative data from the following three large-scale projects: the Child Health and Migrant Parents in Southeast Asia project (CHAMPSEA) [5,27,28,46,47], the Effects of migration on Children and the Elderly Left Behind in Moldova and Georgia (CELB-MD/GE) [4,8,33,35] and the surveys among secondary school children in Ghana, Nigeria and Angola 2010/2011 [24,30]. The CHAMPSEA consisted of cross-sectional surveys of two age groups (pre-school and elementary school) of LBC in Indonesia, the Philippines, Thailand and Vietnam. The CELB-MD/GE is a nationwide household survey applied in Eastern Europe targeting labour migrants originating from Moldova and Georgia.

#### 3.2.6. Risk of Bias within Studies

Overall, we found a moderate risk of bias for most studies, however, many authors failed to report participation/response. Considering the predominance of cross-sectional studies, exposures of interest could not be measured prior to the outcomes allowing only the study of associations. With respect to the longitudinal studies, none reported about loss to follow-up, which considerably weakens their study quality.

### 3.3. Results of Individual Studies

To investigate the impact of international parental migration on LBC in the context of transnational family characteristics, we first grouped and analysed studies based on their region of origin. Additionally, we report on gender and age characteristics.

#### 3.3.1. Americas

Three studies addressed parental migration from Mexico to the USA. Heymann, Flores-Macias [44] and Lahaie, and Hayes [29] analysed data from the same Mexican household survey and found higher emotional and behavioural problems among LBC than non-LBC. Aguilera-Guzman and de Snyder [42] applied mixed methods to investigate stressors and compensators among LBC by using The Scale of Stress Associated with Father’s Physical Parental Absence due to International Migration (SSA-FPAIM), ranging from 0 to 160. The authors found that children of migrants were more vulnerable to stress than children of non-migrants (SSA-FPAIM mean 74.4, SD = 31, Cronbach’s alpha = 0.91) [26,42]. Pottinger [36] observed anger (45%) and a fair of insecurity (77%) among LBC in Jamaica. Meanwhile, Nguyen [41] found higher but not significant Cognitive Development Assessment CDA (range 0–15, CDA-LBC = 9.68, CDA-NLBC = 9.67) and Peabody Picture Vocabulary Test PPVT (range 0–125) scores (PPVT-LBC = 61.96, PPVT-NLBC= 58.54) among LBC in Peru. These tools were used as a measure of cognitive ability among LBC [41].

#### 3.3.2. South Asia

Two studies were done in Sri Lanka [34,45] and both found a significant negative association between parental migration and mental health of LBC. The comparative cross-sectional study of Wickramage and Siriwardhana [34] showed that more than 30% of LBC had mental health problems as well as worse mental health outcomes compared with children residing with their parents. A longitudinal study [41] found that LBC have delayed cognitive development in India (score PPVT-LBC = 52.40, PPVT-NLBC = 59.65).

#### 3.3.3. South-East Asia

Adhikari and Jampaklay [26] found no association between parental migration and mental health outcomes of LBC in Thailand. However, a strong negative impact of fathers’ absence on mental health and well-being was observed in Indonesia and Vietnam [27,40,47] (see Table 1). Asis [43] claimed that, in the Philippines, LBC have better well-being outcomes (social anxiety score: range 0–12, mean, SD; LBC = 4.77, SD = 2.45; NLBC = 5.18 SD = 2.30) than those residing with their parents. Mordeno and Gallemit [39] examined the role of personal psychological resources (PPRs) in the well-being of LBC and found that the reach PPR moderated the negative impact of migratory separation. In contrast, the studies of Jordan and Graham [28], Graham and Jordan [5] and Smeekens and Stroebe [31] reported negative associations of parents‘ labour migration on the happiness and resilience of LBC. Especially in Indonesia, the Philippines and Vietnam, children of migrant mothers had worse psychological health and well-being outcomes when compared with children of non-migrant parents or to children with a migrating father. Jampaklay and Vapattanawong [46] focused on resilience of children of migrant fathers showing they (40.8%) were more resilient than children of non-migrants (30.5%) and benefited from the father‘s international employment migration, which is the most common type of migration in Thailand.

#### 3.3.4. Eastern Europe

For the Moldova and Georgia results of the nationally representative survey (CELB/GE), Cebotari and Siegel [8] suggested that LBC had better or no differing health and well-being outcomes than children of non-migrants. A study of Gassmann and Siegel [4] examined a combined well-being index and had similar findings in Georgia (LBC = 90.9%, NLBC = 82.1%), however, the authors found no association for LBC’s well-being in Moldova (LBC = 84.8%, NLBC = 83.9%). Vanore [35] found factors such as caregiving practice and living in the Adjara region to be negatively influential for LBC in Georgia. Tomsa and Jenaro [32] investigated the mental health and coping abilities of LBC in Romania and found significantly higher levels of anxiety (State-Trait Anxiety Inventory STAIC, 40-item, mean LBC = 34.50 SD = 7.31, NLBC = 32.68 SD = 6.16 P = 0.02) and depression (Short Mood and Feelings Questionnaire SMFQ 13-item, mean LBC = SD = 8.76, NLBC = 7.24 SD = 5.15 P = 0.01) among LBC compared with children residing with both parents; yet both groups had similar coping strategies [32]. In the study of Botezat and Pfeiffer [37], Romanian LBC showed a high probability (44.3%) of developing depression. Leskauskas and Adomaitiene [38] investigated self-reported mental health and well-being outcomes among LBC in Lithuania. The authors observed purely negative results in all outcomes, e.g., missing parent (OR = 4.72, *p* < 0.05), and emotional/behavioural problems (OR = 1.71, *p* < 0.05).

#### 3.3.5. African Context

Longitudinal analysis of Ghanaian LBC showed no worse mental health and well-being outcomes compared with non-LBC [7]. The authors investigated transnational family characteristics and self-rated health, happiness, life satisfaction and school enjoyment among public and private school children. The study emphasised the importance of childcare stability and the role of caregivers. LBC were found to be better-off than children from non-transnational families when remaining family members provided good care. However, the authors considered a non-stable family environment, frequent change of caregiver and migrant parents’ divorce as significant risk factors for the well-being of LBC. In another study, Mazzucato and Cebotari [24] observed similar results. In Nigeria, studies showed poorer health outcomes among LBC of divorced parents, older children and in children whose mothers migrate [3]. In a cross-country comparison, Angolan LBC showed worse mental health and well-being outcomes when compared with Ghanaian and Nigerian LBC. Furthermore, good caregiving practices did not mediate the negative associations, while it did in Nigeria and Ghana [30].

#### 3.3.6. Cross-Regional Comparison

Nguyen [41] used panel data of the Young Lives Survey 2007–2009 to investigate the cognitive abilities of LBC aged 5–8 in four different countries—Ethiopia, India, Peru and Vietnam. The author found that cognitive ability test scores were higher among LBC than non-LBC only in Peru, and lower in all other settings, but only significantly in India. The author argued that lower scores are associated with longer migratory separation. By using longitudinal data, this comparative study showed that regardless of the benefits of remittances, children’s cognitive development was delayed due to parental migration across geographically different regions. Wu and Cebotari [13] compared effects in Ghana and in Chinese LBC, concluding that in both settings, LBC are more vulnerable than children who reside with their parents. Fellmeth and Rose-Clarke [12] conducted a systematic literature review on the impact of parental migration on LBC investigating physical and mental health outcomes with 91 studies of the 111 included studies focusing on internal migration in China. Of the 20 studies on international migration, 11 analysed outcomes other than mental health and well-being (e.g., nutrition, physical growth, anaemia, impact of remittances, education). The remaining nine studies are also included in our systematic literature review [12].

### 3.4. Gender Aspects

Gender of LBC and of the migrating parent was an important aspect which was analysed in some studies, although no study focused primarily on this. In Mexico, Aguilera-Guzman and de Snyder [42] investigated the effects of migrating fathers on the psychological health of their offspring within a theoretical framework of stress mediation. They found that in Mexican families, fathers’ migration, which is most common, was not a stress factor for LBC. However, teenage children of both genders had an increased workload due to father’s absence that created social inequality and vulnerability, putting them at higher risk for developing adverse mental health outcomes. In contrast, migrating fathers tended to be associated with favourable well-being outcomes of LBC in Thailand [46]. Yet, a gender-differentiated analysis for Moldova showed more behavioural problems and emotional symptoms among LBC of migrating mothers, as opposed to migrating fathers and to non-LBC [33]. In South East Asian studies, early migration of the mother had a significant negative effect on the mental health of LBC [26,28,31], whereas in Ghana, no differences were found [24]. With respect to the gender of LBC, studies from Ghana, Moldova, Georgia and Romania found that girls in migrant households were generally more vulnerable than boys regardless of which parent migrated [7,8,13,37]. A longitudinal analysis on the well-being of Ghanaian LBC showed that girls were at increased risk to develop adverse psychological health outcomes (declined happiness, life satisfaction, school enjoyment) [7]. The authors suggested that in both African and Eastern European cultures, boys are favoured over girls, causing LB girls to take over more responsibility for the household from the absent parent [8,13].

### 3.5. Age

Some studies examined the age of LBC in the context of parental migration. Cebotari and Mazzucato [3] suggested that the age plays an important role on which impact migrating parents have on children’s wellbeing. The authors found that older children in African transnational families were more vulnerable than younger children. Gassmann and Siegel [4] also considered age as an important predictor for LBC well-being in Moldova and Georgia, claiming that it increases with age. Nguyen [41] emphasised the importance of child development by age 5–8 and argued that leaving children behind at this crucial age delays cognitive development. Aguilera-Guzman and de Snyder [42] argued that teenage LBC are at increased risk to develop stress, behavioural and other mental health disorders. Adolescents in transnational families usually have to take responsibility and perform more routine tasks, which may indirectly increase their vulnerability and lead them to risky behaviour such as alcohol consumption, drug abuse and smoking [8,13,42].

### 3.6. Synthesis of Results

Seven studies assessed mental health through SDQ and TDS. In Angola, Ghana, Indonesia, Lithuania, Nigeria Sri Lanka and Thailand, LBC developed adverse mental health outcomes more frequently than children whose parents did not migrate, while the opposite was observed in the Philippines (see Table 1 and Table 2). However, a separate analysis by migrant parents’ gender showed that migration of fathers is associated with poorer mental health in Indonesia and Vietnam, while the migration of mothers has a stronger negative impact on LBC in Thailand. In Moldova, Vietnam and the Philippines, children of migrant mothers showed better mental health outcomes comparative to both children of non-migrant parents and children of migrant fathers only (Table 1 and Table 2).

### 3.7. Comparative Analysis of Main Outcomes

Studies suggested that mental health and well-being outcomes of LBC differ across regions and sometimes even within regions. To better illustrate the results across regions, we summarised and compared the evidence of individual studies in Table 3, showing the main findings by outcome measure and region.

All studies conducted in the Americas [29,42,44] and South Asia [34,45] showed negative effects of parental migration (higher behavioural problems and mental health disorders, lower well-being and coping abilities) among LBC, while in other regions, results are incongruent. Studies from Eastern Europe showed negative effects in four, no differences in five and positive results in three outcomes among LBC, as shown in Table 3, and we observed a similar pattern in South East Asia, with six positive, seven negative and one inconclusive results. Meanwhile, in Africa, LBC seem to be negatively affected in seven outcomes, but no difference was observed in four outcomes.

## 4. Discussion

The results of this systematic literature review suggest that effects of parental migration on mental health and well-being of LBC are not always negative but vary from negative to positive, depending on age and gender of LBC, gender of the migrating parent, family norms, caregiving practice as well as other family characteristics. Even though many children in many low- and middle-income countries are affected by international parental migration, its complex and long-lasting effects are not well explored.

We found an unequivocally negative impact of migratory separation on LBC only in two regions—the Americas (Jamaica, Mexico, Peru) and South Asia (India, Sri-Lanka). Eastern European and South East Asian countries have a comparable migration profile with respect to increasing tendency of labour migration, but parents’ migration affected children differently.

In South East Asia, findings were somewhat contradicting, ranging from strong negative to positive effects with declined happiness and lower abuse among LBC. In Eastern European countries, results ranged from no effects to even positive effects on LBC in Moldova and Georgia, and negative effects in Romania and Lithuania. Most of the studies conducted in these regions analysed data from the same projects, demonstrating a lack of diversified research. In Africa, results ranged from no effects in Ghana and Nigeria to negative effects in Angola. Generally, most of the studies addressed only the short-term impact of parental migration on LBC. For each region, we only found studies conducted in a few countries (e.g., only India and Sri-Lanka in South Asia and Jamaica, Mexico and Peru in the Americas), making it difficult to generalise findings for entire regions. Inconsistent results may partly be explained by the heterogeneity of study characteristics such as the reported outcome measures. Although some studies used the SDQ to measure child mental health, authors applied different versions, e.g., for younger children through caregivers and for older children with self-evaluation.

An analysis with respect to transnational family characteristics such as family arrangement, role of the mother and father, gender and age of LBC showed culture-associated differences across regions and sometimes even within regions. However, parental migration effects tended to be more similar among LBC from the same region. For example, Mazzucato and Cebotari [24] argued that in African culture, child fosterage is a socially accepted norm and as a result, some children may have caregivers different from their parents, regardless of parental migration. In South East Asia and the Americas in contrast, the nuclear family is the most common form of family arrangement. This difference may be another explanation for variance in mental health and well-being outcomes among children in transnational and non-transnational families across regions. In the literature on transnationalism, the childhood of LBC is often referred to as “transnational childhood” [48]. Our study showed that transnational childhood is a complex phenomenon and family characteristics play a crucial role.

Several studies included in our analysis considered availability of remittances when examining transnational family characteristics. Some authors compared well-being outcomes of LBC and non-LBC in terms of presence or absence of remembrances and found no difference between these groups in the African and Eastern European context [3,8]. The authors suggested that remittances from migrating parents were scarce and were used to address the family member’s basic needs without affecting children’s well-being [3,8,29,44]. Heymann and Flores-Macias [44] claimed that although a migrating parent is the main economic contributor to the family, migratory separation negatively effects not only mental health and well-being of LBC but also their education. In contrast, other authors argued that parent’s labour migration may positively affect educational and health outcomes if remittances are spent accordingly [26,27]. For example, in South East Asia, Graham and Jordan [27] found that LBC from wealthier households tended to have better well-being outcomes than LBC from poorer families. Transnationalism, however, created physical and emotional distance and, as De La Garza [49] argued, family disintegration was the most adverse effect of it.

We found that caregiving practices were another potential explanation for differences in child well-being. Studies included in our analysis showed that children of migrating parents were mostly left in the care of extended family members. Usually, when only one parent migrated, the remaining parent took responsibility for the children. When both parents migrated, typically grandparents were caregivers who often needed care themselves, further complicating the caregiving practices. Our study found lower well-being among children whose primary caregivers were not their parent (e.g., grandparent, other relative) as opposed to children who were taken care of by their parent(s) [4,30]. Moreover, in the study of Vanore and Mazzucato [33], LBC reported violence and verbal abuse from their caregivers. Often, LBC in transnational families were obliged to take over responsibilities of absent parents. Depending on the setting, this included farm work, house work, cooking and care for siblings, among others. These additional demands on the children may increase the negative impact of migratory separation. For example, children who lived in crowded households and with many siblings showed higher vulnerability than children who did not have many siblings in the household [4]. In Ghana, fathers tended to demand much of LBC, causing low levels of life satisfaction and happiness [13].

Several studies included in our analysis considered living conditions when examining transnational family characteristics. Good housing conditions were found to be positively associated with well-being outcomes of LBC in Georgia, while no difference was found in Moldova [4]. Living in urban areas tended to be beneficial for LBC in terms of housing and access to modern communication sources (e.g., mobile phone), but detrimental for health outcomes [4]. Mazzucato and Cebotari [30] found a mediating effect of good living conditions among LBC in Ghana, but no effect in Nigeria and Angola. Again, varying results demonstrate the complexity of transnational family characteristics and their effects on child mental health and well-being.

Cultural diversity and different family arrangements can be the reason for gender-associated differences across countries. For example, in Ghana and Mexico, fathers’ migration was the most common type of migration and mothers took full responsibility for the care of LBC [13]. This is a “family norm” for patriarchal societies, where the husband holds more power and is responsible for the breadwinner of the family. The increasing trend of migrating mothers, also called “feminisation of labour migration” [6,50,51] may shift this paradigm. While this transformation may affect children in different ways, the issue is not well addressed. We encourage further research in this area.

Furthermore, we explored how the age of LBC was related to their mental health and well-being. Depending on age, children responded differently to migratory separation. However, most studies that targeted children under the age of ten asked caregivers instead of children, which may have led to bias.

Congruent with our findings, some scholars have suggested that LBC may also be affected in other areas of their life, such as physical health and education [12]. Studies investigating the effect of remittances on LBC suggested that economic benefits of parental migration may lead to materialism which is indirectly associated with adverse well-being outcomes [52]. Children growing up with a focus on possessions rather than on community or time spent together have poorer well-being [52,53]. On the other hand, Antman [54] sees fathers’ Mexico–US migration and international parental migration in general as a way to improve education attainment of LBC, especially of girls. Authors observed similar results among LBC in the Philippines [55,56], Tajikistan [57], Morocco [58] and Moldova [59], while the opposite was observed in Ghana, Nigeria [60] and Georgia [59]. Overall, our analyses showed that multidimensional family characteristics are crucial and should be better explored when examining the impact of migratory separation on LBC, especially in the framework of transnational migration.

Unlike international migration, internal migration in the context of LBC has been extensively studied in China [61,62,63]. There, LBC seem mostly affected negatively by rural–urban migratory separation [42,62,63,64], while our results on international migration show positive and negative effects. As most evidence on LBC comes from China, the literature on internal migration is often associated with Chinese migration, which cannot be generalised to a global context. Hence, we encourage further research on the impact of international employment migration on LBC in countries sending labour migrants.

This systematic literature review investigated the effect of parental migration on mental health and related well-being outcomes of LBC based on the country of origin, gender and age. We found that most studies did not separate internal rural–urban migration from international migration when reporting the impact of parental migration on LBC. The results, however, differ significantly from each other. Even though individual studies often identified culture, gender and duration of migration to be the factors with most influence on LBC, this evidence has not been synthesised in the context of international parental migration. By focusing on international migration and transnational family characteristics, we present a detailed yet comprehensive analysis.

Several limitations of this systematic review of the literature should be mentioned. First of all, the included studies varied in terms of study design, sample size, age group, tools used to measure outcomes and statistical methods applied. Studies were not comparable enough either to perform a meta-analysis or to strictly evaluate the risk of bias across articles. Our quality assessment has shown some risk of bias in different domains, e.g., selection of participants, clarity, validity and reliability of outcome measures. We used broad search terms to cover all possible regions from where parents migrate internationally; therefore, we might have missed relevant studies from countries that use specific local terms to describe children who remain in their countries of origin while their parents migrate to work in another country. Additionally, we only included studies published in English, and, as a consequence, may have missed relevant articles in other languages. Most of the studies included used a cross-sectional design and can therefore not claim causal relationships between parental migration and health outcomes of LBC.

Most of the included studies come from the fields of sociology, anthropology and transnationalism. However, left-behind family members are not well explored in migration research. Moreover, studies in this area conducted by public health researchers are lacking completely. We highly recommend public health scientists to emphasise the health and well-being outcomes of LBC. In order to implement effective policies, we need more research and evidence not only on the impact of parental migration on LBC, but also on the needs of those children and their families.

## 5. Conclusions

Our study shows that the impact of parental migration on LBC is not purely negative and very much depends on the characteristics of the transnational families. Gender and age of the children, gender of the migrant parent, stability of care, parental divorce and living conditions all influence children’s mental health and well-being. Overall, only studies conducted in the Americas and South Asia observed purely negative effects of migratory separation. In some countries, left-behind children showed abnormal Strengths and Difficulties Questionnaire scores (e.g., Angola, Ghana, Indonesia, Lithuania, Nigeria Sri Lanka) and reported higher levels of depression and loneliness than children who did not live in transnational families, while in other countries, LBC tended to be better-off than non-LBC. Our analysis shows gender-associated differences across regions, e.g., in some South East Asian countries, migration of mothers tended to be negative for the mental health of LBC, whereas in some African countries, the migrating parent’s gender made no difference. We found that in the African and Eastern European regions, girls in migrant households tend to be more vulnerable than boys, regardless which parent migrated. LBC whose parents were divorced, who did not live in a stabile family environment, who changed their caregivers frequently and who lived in a crowded household tended to have worse well-being outcomes than LBC who lived in stable family environments. Our findings suggest that remittances sent by migrating parents were usually used up by the essential needs of family members and did not contribute significantly to a better socioeconomic status or well-being of LBC.

Our analysis revealed that the data within and across regions are hardly comparable, identifying LBC research in the context of international migration as a research gap. Hence, we encourage scholars, especially from the field of public health to collaborate with other disciplines and to generate evidence on LBC focusing on gender and age aspects.

## Figures and Tables

**Table 1:** Abnormal Strengths and Difficulties Questionnaire (SDQ) scores by country and migration status.

Country		Study	SDQ Non-LBC (Control)	Father Migrated		Mother Migrated	
				SDQ	Odds Ratio [95% CI]	SDQ	Odds Ratio [95% CI]
**Indonesia**		[27]	25.4	42.5	2.19 [1.50–3.20]	31.7	1.35 [0.97–1.89]
**Moldova**	male LBC	[33]	12.1	13.1	1.12 [0.71–1.72]	10.8	0.89 [0.50–1.49]
**Moldova**	female LBC	[33]	16.7	13.5	0.77 [0.49–1.17]	15.6	0.93 [0.57–1.45]
**The Philippines**		[27]	25.6	18.9	0.68 [0.48–0.95]	16.4	0.57 [0.29–1.04]
**Thailand**		[27]	11.3	11.1	0.99 [0.65–1.49]	-	-
**Thailand**		[26]	11.0	13.4	1.26 [0.87–1.82]	22.8	2.40 [1.73–3.36]
**Vietnam**		[27]	24.9	33.5	1.53 [1.05–2.20]	15.2	0.55 [0.36–0.81]
				**Any parent migrated**			
**Indonesia**		[40]	21	28.4	1.49 [0.66–3.53]	-	-

**Table 2:** Total Difficulties Score (means). Higher score indicates higher psychological distress; analysis of variance (ANOVA) [24,30] and chi-squared test [38] were used for comparisons.

Country	Study	TDS Non-LBC	TDS LBC	*p* Value
**Angola**	[30]	13.0	16.1	<0.001
**Ghana**	[30]	11.3	12.1	<0.05
	[24]	11.3	11.5	not significant
**Lithuania**	[38]	10.2	11.4	<0.05
**Nigeria**	[30]	10.9	11.8	<0.001

**Table 3:** Mental health and well-being outcomes of left-behind children (LBC) in comparison to the children of non-migrant parents.

	Higher among LBC	No Difference	Lower among LBC
**positive outcomes**			
**Africa**		Happiness [7], satisfaction [3], school enjoyment [46], self-rated health [7]	satisfaction [13], school enjoyment [28], self-rated health [3], well-being [24,30], cognitive ability [41]
**Eastern Europe**	self-rated health [8], well-being [4]	coping [32], well-being [4]	
**Americas**			self-esteem [36], coping [42], well-being [29,44], cognitive ability [41]
**South Asia**			cognitive ability [41]
**South East Asia**	well-being [43], personal psychological resources [39]		happiness [5,31,43,46,47], cognitive ability [41], well-being [27,28,47]
**negative outcomes**			
**Africa**	behavioural problems [24,30], mental health disorders [24,30]		
**Eastern Europe**	anxiety/stress [32,37], behavioural problems [33,38], emotional problems [38], depression [32,37]	anger [32], behavioural problems [4], mental health disorders [4,33,35]	mental health disorders [4]
**Americas**	behavioural problems [13,36]		
**South Asia**	behavioural problems [45], mental health disorders [34]		
**South East Asia**	anxiety/stress [31], loneliness [31,40], mental health disorders [27], behavioural problems [40]	mental health disorders [26,27]	abuse [43], anxiety/stress [43], loneliness [31], mental health disorders [26]

## Appendix A

**Table A1:** Characteristics of included studies by country and age.

Country	Study Type	Age	Sample N	Outcome	Risk of Bias ^1^	Source
Mexico	cross-sectional	0–15	1509 (households)	Emotional health, behavioural problems	B (9/14)	[29]
Mexico	mixed	0–15	1509 (households)	Well-being	B (9/14)	[44]
Mexico	mixed	11–14	310	Stressors, compensators	B (8/14)	[42]
Sri Lanka	cross-sectional	0–18	820	Mental health	B (8/14)	[34]
Sri Lanka	mixed	5–10	253	Mental health	B (9/14)	[45]
Thailand	mixed	9–11	496	Subjective well-being, resilience	A (10/14)	[46]
Thailand	cross-sectional	3–5, 9–11	519 LBC, 511 NLBC	Mental health	B (8/14)	[26]
Indonesia	cross-sectional	11–15	359 LBC, 270 NLBC	Emotional and behavioural problems, loneliness	B (7/14)	[40]
Indonesia, The Philippines, Thailand, Vietnam	cross-sectional	0–12	3876 (households)	Child well-being SDQ	B (8/14)	[27]
Indonesia, The Philippines	mixed	0–12	1010	Subjective well-being - Self-reported happiness	B (9/14)	[5]
Indonesia, The Philippines	mixed	9–11	Indonesia: 513 The Philippines: 500 (households)	Well-being; general happiness	B (8/14)	[47]
Indonesia, The Philippines, Vietnam	cross-sectional	9–11	1498	Psychological well-being	B (9/14)	[28]
The Philippines	mixed	10–12	1443	Well-being: incidence of abuse, emotional health	B (9/14)	[43]
The Philippines	cross-sectional	11–17	466	Mental health, well-being, parent-child relationship	B (7/14)	[39]
The Philippines	cross-sectional	13–18	205	Missing parents, perceived stress, loneliness, coping	B (7/14)	[31]
Moldova	cross-sectional	4–17	1979	Psychosocial health SDQ	B (8/14)	[33]
Georgia	cross-sectional	4–17	1282	Psychosocial health SDQ	B (8/14)	[35]
Moldova, Georgia	cross-sectional	5–17	Moldova: 3571 Georgia: 4010 (households)	Multidimensional well-being	B (7/14)	[4]
Romania	cross-sectional	11–15	279 LBC, 1142NLBC	Psychological well-being	B (9/14)	[37]
Romania	cross-sectional	12–15	163 LBC, 163 NLBC	Anxiety, anger, depression, Coping	B (8/14)	[32]
Moldova, Georgia	cross-sectional	10–18	Moldova: 1601 Georgia: 1193	Child Health, well-being	B (8/14)	[8]
Lithuania	cross-sectional	10–19	1292	Emotional and behavioural problems	B (7/14)	[38]
Ghana, Nigeria	cross-sectional	11–18	Ghana: 2760 Nigeria: 2168	Self-rated health emotional wellbeing	B (8/14)	[3]
Ghana	cross-sectional	11–21	2760	Self-reported psychological health, well-being	B (8/14)	[24]
Ghana, Nigeria, Angola	cross-sectional	11–21	Ghana: 2760 Angola: 2243 Nigeria: 2168	Self-reporting, psychological well-being	B (8/14)	[30]
Ghana	longitudinal	12–21	741	Self-rated health, happiness, life satisfaction	A (11/14)	[7]
Jamaica	case-control	9–10	27 LBC, 27 NLBC	Psychological difficulties, behaviour, emotional well-being	B (6/12)	[36]
Ethiopia, India, Peru, Vietnam	longitudinal	5–8	7725	Cognitive ability	B (9/14)	[41]
China, Ghana	cross-sectional	11–20	Ghana:1622	Child well-being: life satisfaction, resilience, vulnerability	B (7/14)	[13]
International	SR and meta-analysis	0–19	106167 LBC, 158800 NLBC	Mental health, other	A (8/8)	[12]

^1^ Risk of bias ABC: A—low, B—moderate, C—high risk; study quality assessment scores in brackets.
